# Prognostic Significance of Nomogram and T-Score in Locally Advanced Cervical Cancer Patients Treated with Curative Chemoradiotherapy and Image-Guided Brachytherapy: A Single-Center Retrospective Study

**DOI:** 10.3390/diagnostics15172142

**Published:** 2025-08-25

**Authors:** Kamuran Ibis, Can Ilgin, Leyla Suncak, Canan Koksal Akbas, Deniz Bolukbas, Mustafa Denizli, Abdulmunir Azizy, Begum Yilmaz, Seda Guler Ozben, Ayca Iribas Celik, Nezihe Seden Kucucuk, Inci Kizildag Yirgin

**Affiliations:** 1 Department of Radiation Oncology, Institute of Oncology, Istanbul University, 34093 Fatih, Istanbul, Türkiye; canilgin@hotmail.com (C.I.); bolukbasdnz@gmail.com (D.B.); mustafadenizli_95@hotmail.com (M.D.); yilmazbgmm@gmail.com (B.Y.); sedaozben@gmail.com (S.G.O.); aycairibas@hotmail.com (A.I.C.); seden.kucucuk@gmail.com (N.S.K.); 2 Department of Medical Physics, Institute of Oncology, Istanbul University, 34093 Fatih, Istanbul, Türkiye; leylasuncak@gmail.com (L.S.); canankksal@gmail.com (C.K.A.); 3 Department of Medical Oncology, Institute of Oncology, Istanbul University, 34093 Fatih, Istanbul, Türkiye; munir.azizy@gmail.com; 4 Department of Radiology, Institute of Oncology, Istanbul University, 34093 Fatih, Istanbul, Türkiye; inci.kizildag@gmail.com

**Keywords:** brachytherapy, cervical cancer, radiotherapy, overall survival, local recurrence

## Abstract

**Objective**: To investigate the survival prediction probability of the nomogram from retro-EMBRACE and the T-score in patients with locally advanced cervical cancer (LACC). **Materials and Methods**: A total of 204 patients with LACC who underwent curative chemoradiotherapy and brachytherapy (BT) between 2010 and 2021 were included in our single-center retrospective study. Clinical records, examinations, and magnetic resonance images (MRI) before and after external beam radiotherapy (EBRT) were retrospectively reviewed to obtain information on age, tumor size, parametrial involvement, ureteral involvement, bladder invasion, uterine involvement, high-risk clinical target volume at the first brachytherapy application, lymph node involvement, vaginal involvement, recurrence, metastasis, and last follow-up. The 5-year overall survival probabilities of the patients were determined by nomogram. T-score was calculated separately at diagnosis (TS_D_) and brachytherapy (TS_BT_), and their effects on local recurrence-free survival, disease-free survival, and overall survival were analyzed. **Results**: The median age was 52 (29–89). The 5-year survival rate of the patients was calculated to be 90.18%. The median nomogram’s survival estimate for 60 months was 70.35% (20.9–87.1). The median TS_D_ and TS_BT_ were 5.5 (1–16) and 1 (0–6), respectively. According to the multivariate Cox regression models, TS_D_ (HR = 1.203, 95% CI 1.021–1.417, *p* = 0.024) was significantly associated with local recurrence-free survival. **Conclusions**: This study demonstrated that the nomogram’s predictions for 60-month overall survival are underestimates. Prognosis can be estimated using the TS_D_, which can be easily obtained with a clinical examination and detailed MRI examination.

## 1. Introduction

Cervical cancer is one of the most preventable, screenable, and curable malignancies due to its well-defined etiology, population screening programs, and developments in chemoradiotherapy (CRT), immunotherapy, and surgery [1,2]. Nevertheless, cervical cancer causes a severe disease burden, especially in developing countries [3]. After the diagnosis of cervical cancer, the prediction of prognosis in cervical cancer patients is essential for guidance of treatment [4]. Concurrent CRT and brachytherapy (BT) is the treatment of choice for locally advanced cervical cancer (LACC) [5]. Although more advanced techniques are being used to deliver external beam radiotherapy (EBRT), BT remains a crucial component for achieving optimal local control and long-term survival [6,7].

Performance status is one of the primary determinants of a patient’s compliance with oncological treatment and tolerance to its toxicity. One of the scales used to assess performance status is the Karnofsky performance scale (KPS), developed in 1948 [8]. It was divided into 10 levels based on patient condition and degree of self-care, with 10 points assigned for each level, totaling 100 points. A score below 60 indicates poor health and a poor quality of life. It is widely used in the assessment of cancer patients [9].

With the advances in technology, as in EBRT, BT has shifted from two-dimensional (2D) treatment methods to three-dimensional (3D) treatment methods. Today, image-guided adaptive BT (IGABT) using MRI (preferred) or computed tomography (CT) is the new gold standard in BT for cervical cancer [10]. The transition from 2D to 3D-IGABT in cervical cancer was developed with the establishment of the GEC-ESTRO-GYN working group in 2000, comprising clinicians from several leading European centers. The GEC-ESTRO-GYN working group established a network in 2005 to enhance cooperation among centers interested in IGABT. In 2008, the GEC-ESTRO GYN group initiated the “International study on MRI-based Brachytherapy in locally Advanced Cervix cancer” (EMBRACE) study to evaluate the results of IGABT in a multicenter setting.

The first EMBRACE study (EMBRACE-I) was a prospective observational study of CRT and MRI-based IGABT. Quality assurance was performed to ensure uniform target definition and dose reporting of IGABT according to the GEC-ESTRO recommendations; however, institutions varied in their practices regarding EBRT and BT techniques and dose prescription. The study, initiated in 2008, was completed by the end of 2015, involving 1416 patients. In 2010, the GEC-ESTRO-GYN network initiated Retro-EMBRACE, a retrospective study of patients treated with CT- or MRI-based IGABT before the start of EMBRACE-I. The Retro-EMBRACE study, which examined 711 patients, demonstrated that IGABT yielded excellent local and pelvic control [11].

Sturdza et al. aimed to establish a nomogram for the prediction of overall survival (OS) in patients with LACC who underwent definitive chemotherapy, including IGABT. They constructed the first nomogram to predict OS in patients with LACC treated with IGABT, including FIGO stage, high risk-clinical target volume (HR-CTV) in first fraction BT, corpus involvement, chemotherapy use, total treatment duration, age at diagnosis, and lymph node status [12].

Stage is an essential factor in determining the prognosis of LACC. However, information such as proximal or distal parametrial invasion, unilateral or bilateral parametrial involvement, and uterine corpus involvement, which can be obtained by clinical examination and magnetic resonance imaging (MRI), is not included in the staging. Lindegaard et al. have worked to enhance the performance of a comprehensive yet straightforward tumor score for determining prognosis. They scored the involvement of the cervix, left parametrium, right parametrium, vagina, bladder, ureter, rectum, and uterine corpus according to an ordinal scale of 0 to 3 points, resulting in a total tumor score (T-score). They analyzed 400 consecutive patients with LACC treated with CRT and IGABT between 2005 and 2018. Consequently, the T-score was established as a prognostic tool for survival and local control [13]. Lindegaard et al. published another study in 2022, in which they enrolled 1318 patients eligible for the T-score who were treated with CRT followed by MRI-guided BT in the EMBRACE I trial. The aim was to validate the performance of the T-score using the multicenter EMBRACE I trial and to evaluate the prognostic implications of T-score regression achieved during initial CRT [14].

However, data on the regional adaptability of these scales for other patient populations, as well as their predictive performance on oncological outcomes for these patients, are limited. Although MRI-based IGABT is the standard treatment for BT of cervical cancer, CT-based IGABT is frequently used because cervical cancer is often seen in countries with limited economic resources.

In this study, we evaluated the relationship between pre- and post-treatment T-scores, T-score regression patterns, and the nomogram by Sturdza et al. in conjunction with 60-month survival estimates and oncological outcomes of patients with LACC treated with CT-based IGABT at our institute.

## 2. Materials and Methods

A total of 204 patients with LACC who were treated with curative CRT and CT-based 3D-IGABT between 2010 and 2021 were included in our single-center retrospective study. Of the patients, 192 (94.1%) were staged by both PET/CT and MRI, and 12 (5.9%) were staged by MRI. All patients were treated using 3D-CRT or IMRT/VMAT techniques in EBRT followed by intracavitary HDR CT-based 3D-IGABT.

The sociodemographic and clinical features, including age, Karnofsky performance scale (KPS), hemoglobin level, histology, FIGO stage, presence of lymphadenopathy, chemotherapy, radiotherapy (dose, fraction and volume information, BT application), treatment duration, and outcomes during follow-up, were recorded from both physical and digital patient records.

Clinical records, examination findings, and MRIs before and after EBRT were retrospectively examined. According to the study published by Lindegaard et al. in 2019, the MRIs of the patients taken before CRT, patient records, and clinical drawings were examined [13]. Eight anatomical locations were scored according to an ordinal scale [uterine cervix (0 point: Not present, 1 point: ≤20 mm, 2 points: >20–≤40 mm, 3 points: >40 mm), right/left parametrium (0 point: Not involved, 1 point: Proximal, 2 points: Distal, 3 points: Pelvic wall), bladder (0 point: Not involved, 1 point: Bladder wall, 2 points: Bullous edema, 3 points: Mucosa involved), ureter (0 point: Not involved, 1 point: Unilateral, 2 points: Bilateral), rectum (0 point: Not involved, 1 point: Mesorectum, 2 points: Rectal wall, 3 points: Mucosa involved), uterine corpus (0 point: Not involved, 1 point: Lower 1/3, 2 points: Middle 1/3, 3 points: Upper 1/3), vagina involvement (0 point: Not involved, 1 point: Upper 1/3, 2 points: Middle 1/3, 3 points: Lower 1/3)] [13]. Thus, T-scores were calculated before EBRT, and four groups were formed as suggested (group I: 0–3 points, group II: 4–5 points, group III: 6–7 points, group IV: >7 points).

Later, according to a study published by Lindegaard et al. in 2022, the same procedure was performed after CRT [14]. Thus, the T-score was separately calculated at diagnosis (TS_D_) and brachytherapy (TS_BT_) by summing the points obtained from the eight locations on both occasions. As reported in the same study, a tumor regression pattern was created using T-score points obtained at the time of diagnosis and before brachytherapy. According to the regression pattern, the data were grouped into three categories: Low (TS_D_ ≤ 5, L), High–Low (TS_D_ > 5 but TS_BT_ ≤ 5, HL), and High–High (TS_D_ and TS_BT_ > 5, HH).

**Figure 1:**
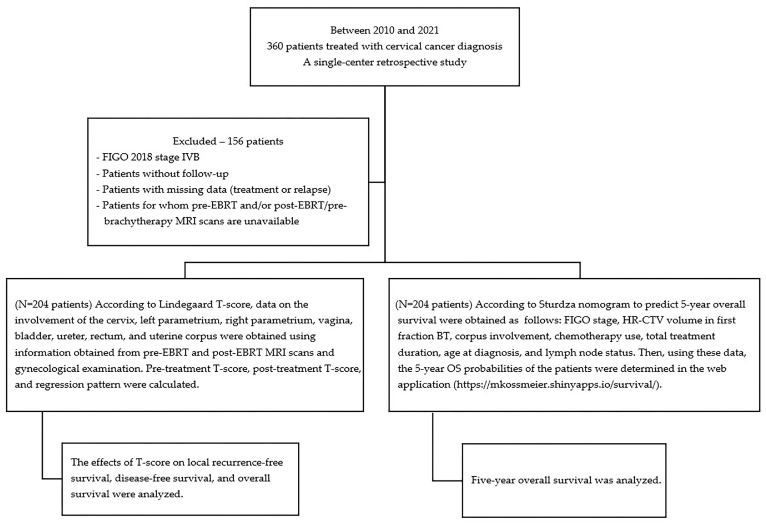
Schematic diagram of the study design.

FIGO stage (FIGO 2009), HR-CTV volume (cc) in the first BT fraction, corpus involvement, concurrent use of chemotherapy, total treatment duration, age at diagnosis, and lymph node involvement data were obtained, which Sturdza et al. used to create the nomogram. Then, using these data, the 5-year OS probabilities of the patients were determined in the web application developed by Sturdza AE et al. (https://mkossmeier.shinyapps.io/survival/ (accessed on 19 January 2025)) [12] (Figure 1).

The time from the biopsy date to the last follow-up or death was defined as OS; the time until the local recurrence was defined as local recurrence-free survival (LRFS); and the time until the local, regional, or distant metastasis was defined as disease-free survival (DFS), whichever came first. The numerical variables were presented with median, interquartile range, minimum, and maximum values. The categorical variables were presented with frequencies and percentages. The survival curves for oncological outcomes are compared using the log-rank test for the equality of survivor functions, and the results are reported with *p*-values. Univariate and multivariate analysis was performed by Cox regression analysis. A log-rank test was used for Kaplan–Meier curves. The univariate and multivariate Cox regression models were presented using hazard ratios (HRs) with 95% confidence intervals. The AUC values of ROC curves were reported with 95% confidence intervals. A *p*-value less than 0.05 was considered statistically significant. Statistical analyses were performed using Stata 15.1 (StataCorp, 4905 Lakeway Drive, College Station, TX 77845, USA).

This retrospective study was approved by the Clinical Research Ethics Committee of Istanbul Faculty of Medicine, Istanbul University (2024/1306).

## 3. Results

A total of *n* = 204 patients with a median age of 52 (IQR = 16, ranging between 29 and 89) were included in this study. The median follow-up duration for patients was 99 months (IQR = 51), ranging from 16 to 172 months. The majority of patients had a KPS score of 100 (*n* = 134, 65.69%), followed by 90 (*n* = 59, 28.92%) and 80 (*n* = 10, 4.90%), with only one patient having a KPS score of 70. The majority of patients had squamous cell carcinoma (*n* = 186, 91.18%), while other histologies included adenocarcinoma (*n* = 12, 5.88%), adenosquamous carcinoma (*n* = 2, 0.98%), and serous and mucinous papillary carcinoma (each *n* = 1, 0.49%). Lymphadenopathy was present in 92 patients (45.10%), with the location of lymphadenopathy primarily being pelvic (*n* = 79; 38.73%) or pelvic and para-aortic (*n* = 11; 5.39%) in most cases. Only two patients (0.98%) had lymphadenopathy limited to the para-aortic region. The patients were dichotomized according to the FIGO 2018 stages, with *n* = 96 (47.06%) patients classified as stage I-II and *n* = 108 (52.94%) patients classified as stage III-IV.

While only two patients (0.98%) received neoadjuvant chemotherapy, the majority of patients (*n* = 200, 98.04%) received concurrent chemotherapy. The majority of patients received cisplatin-based regimens (*n* = 198, 97.06%) and received them every week (*n* = 164, 80.39%). More than half of the patients (*n* = 112, 54.90%) received conformal radiotherapy, while 92 patients (*n* = 45.10%) received IMRT. The median EBRT dose was 50 Gy (ranging from 45 to 64.4 Gy) for the cervix and 50 Gy (ranging from 45 to 54 Gy) for the pelvic lymph nodes, respectively. The median fraction number was 25, ranging from 23 to 35. The median number of BT fractions and fraction dose were 4 (ranging from 2 to 6) and 6 (ranging from 5 to 8.5), respectively. More than half of the patients (*n* = 106, 51.96%) had an HR-CTV volume exceeding 30 cc, while 98 patients (48.04%) had a volume of 30 cc or less. The median EQD2 (equivalent dose in 2 Gy fraction) of HR-CTV D90 for α/β =10 was 81.65 (IQR = 5.85, ranging from 70.1 to 97.2). The treatment period was 80 days in 48.53% (*n* = 99) of the patients and longer than 80 days in 51.47% (*n* = 105).

The median TS_D_ was 5.5 (IQR = 3, range 1–16). The distribution of TS_D_ groups was as follows: TS_D_ 0–4, 64 (31.4%); TS_D_ 5–6, 63 (30.9%); TS_D_ 7–9, 56 (27.5%); and TS_D_ > 9, 21 (10.3%). The median TS_BT_ was 1 (IQR = 1, range 0–6). The distribution of TS_BT_ groups was as follows: TS_BT_ 0–4, 198 (97.1%); TS_BT_ 5–6, 6 (2.9%). Half of the patients exhibited a regression pattern of L (*n* = 102, 50.0%), and 100 patients (49.0%) had HL; only two patients had HH. Patient and treatment characteristics are presented in Table 1.

Pre-EBRT MRI images, post-EBRT/pre-BT MRI images, and examination data showed that pre-treatment left parametrial involvement was found to be proximal in 113 (55.4%) patients and distal in 15 (7.4%) patients, while post-treatment, proximal involvement was found in 13 (6.4%) patients. Pre-treatment right parametrial involvement was found to be proximal in 87 (42.6%) patients and distal in 5 (2.5%) patients, while post-treatment proximal involvement was found in 2 (2.9%) patients. Pre-treatment, uterine cervix involvement was detected as ≤20 mm in 9 (4.4%) patients, >20–≤40 mm in 67 (32.8%) patients, and >40 mm in 128 (62.7%) patients. Post-treatment, there was no residual tumor in 100 (49%) patients; uterine cervix involvement was detected as ≤20 mm in 9 (4.4%) patients, >20–≤40 mm in 66 (32.3%) patients, and >40 mm in 34 (16.7%) patients. Pre-treatment, bladder wall invasion was detected in five (2.5%) patients and bullous edema in two (1%) patients, while post-treatment, bladder wall invasion was detected in one (0.5%) patient. Pre-treatment, five (2.5%) patients had unilateral ureteral invasion, and one (0.5%) patient had bilateral ureteral invasion, while no involvement was detected after treatment. Pre-treatment, mesorectum invasion was detected in six (2.9%) patients, and rectal wall invasion was detected in one (0.5%) patient, while no invasion was observed after treatment. Pre-treatment, 47 (23%) patients had lower third, 18 (8.8%) patients had middle third, and 7 (3.4%) patients had upper third uterine corpus involvement, while, post-treatment, 6 (2.9%) patients had lower third, 3 (1.5%) patients had middle third, and 2 (1%) patients had upper third involvement. Pre-treatment, vaginal involvement was in the upper third of the vagina in 50 (24.5%) patients, in the middle third of the vagina in 8 (3.9%) patients, and in the lower third of the vagina in 4 (2%) patients, while after treatment, there was involvement in the upper third of the vagina in 4 (2%) patients and in the middle third of the vagina in 1 (0.5%) patient.

**Figure 2:**
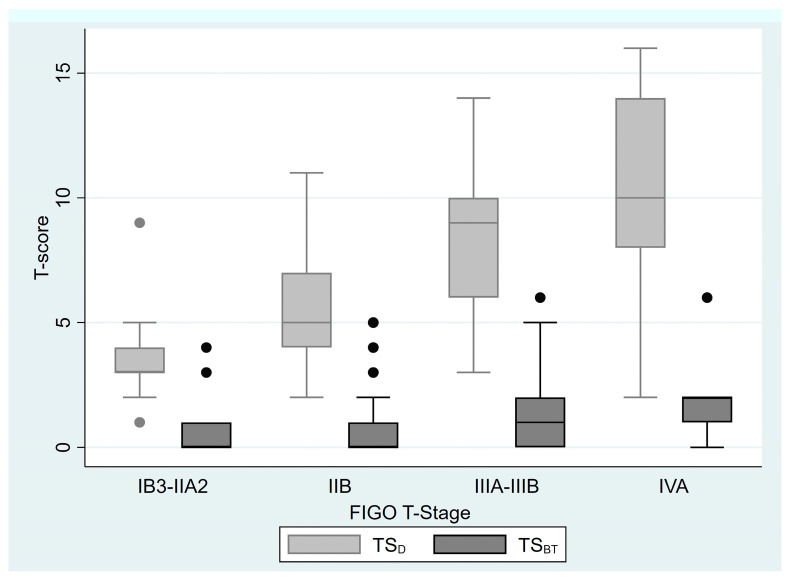
Box plots for TS_D_ and TS_BT_ according to the FIGO T stages. (FIGO: The International Federation of Gynecology and Obstetrics; TS_D_: pre-treatment T-score at diagnosis; TS_BT_: pre-treatment T-score at brachytherapy.)

When staging was performed according to FIGO 2018 T stage, in stage IB-IIA, the median TS_D_ was 3 (1–9), while the median TS_BT_ was 0 (0–4); in stage IIB, the median TS_D_ was 5 (2–11), while the median TS_BT_ was 0 (0–5); in stage IIIA-IIIB, the median TS_D_ was 9 (3–14), while the median TS_BT_ was 1 (0–6); and in stage IVA, the median TS_D_ was 10, while the median TS_BT_ was 2 (0–6). Box plots for TS_D_ and TS_BT_, categorized by FIGO 2018 T stages, are presented in Figure 2.

During follow-up, 21 patients (10.29%) had local recurrence, and 27 patients (13.24%) had a disease-related event. The follow-up of 34 patients (16.67%) resulted in mortality, where the mortality of 20 patients (9.80%) was directly linked to the disease, and the mortality of 9 patients (4.41%) was attributed to other causes. Of the surviving patients (*n* = 170), the majority were disease-free (*n* = 167, 97.65%).

The median OS was 99 months (range, 16–172 months). The 5- and 10-year OS rates were 90% and 79.5%, respectively. The median DFS was 95 months (range, 8–156). The 5- and 10-year DFS rates were 88.3% and 86.4%, respectively. Median LRFS was 96 months (range, 9–172). The 5- and 10-year LRFS were 93.2% and 91.2%, respectively.

In order to evaluate the effects of variables such as age ≥ 65 years, pathology, regression pattern, pelvic and/or para-aortic lymphadenopathy, FIGO 2018 stage group, FIGO 2018 T stage group, pelvic radiotherapy technique, HR-CTV volume ≥ 30 cc, and treatment duration ≥ 80 days on oncological outcome, the survival curves of LRFS, DFS, and OS were compared using the survival function equality test. The results are as follows: Pelvic and/or para-aortic lymphadenopathy (*p* = 0.031) and FIGO 2018 stage group (*p* = 0.024) have a substantial impact on LRFS. The regression pattern has a substantial effect on DFS (*p* = 0.010). FIGO 2018 T stage III-IV disease had a significant impact on OS (*p* = 0.046) (Table 2).

When univariate Cox regression analysis was performed for LRFS, DFS, and OS by including age, KPS score, hemoglobin level, menopause status, pathology, lymphadenopathy, FIGO 2018 T stage group, FIGO 2018 group, 3D-CRT, HR-CTV volume, HR-CTV of EQD2 D90, treatment duration, 60-month estimates by Sturdza et al., TS_D_, TS_BT_, and regression group variables, the results obtained are as follows: LRFS was significantly associated with lymphadenopathy (HR = 3.633, 95% CI 1.035–12.748, *p* = 0.044), FIGO group I-II (HR = 3.413, 95% CI 1.101–10.583, *p* = 0.033), and TS_D_ (HR = 1.184, 95% CI 1.002–1.400, *p* = 0.047). DFS was significantly associated with 60-month estimates by Sturdza et al. (HR = 0.973, 95% CI 0.950–0.997, *p* = 0.028), TS_D_ (HR = 1.193, 95% CI 1.052–1.354, *p* = 0.006), TS_BT_ (HR = 1.297, 95% CI 1.037–1.622, *p* = 0.023), and HL regression pattern compared to L pattern (HR = 3.090, 95% CI 1.298–7.356, *p* = 0.011). OS was significantly associated with 60-month estimates by Sturdza et al. (HR = 0.977, 95% CI 0.957–0.997, *p* = 0.025), TS_D_ (HR = 1.126, 95% CI 1.001–1.265, *p* = 0.048), and TS_BT_ (HR = 1.339, 95% CI 1.096–1.637, *p* = 0.004) (Table 3).

Multivariate analysis was performed for LRFS, DFS, and OS by including variables found to be significant in univariate analysis. According to the multivariate Cox regression model, an increase in TS_D_ (HR = 1.203, 95% CI 1.021–1.417, *p* = 0.027) and the presence of lymphadenopathy (HR = 3.968, 95% CI 1.128–13.957, *p* = 0.032) were found to increase the risk of local recurrence (*p* = 0.0091) (Table 4).

**Figure 3:**
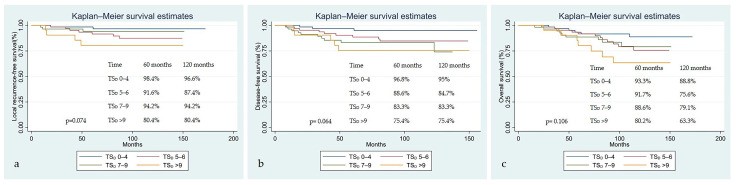
Survival according to pre-treatment T-score at diagnosis (TS_D_) groups: (**a**) local recurrence-free survival; (**b**) disease-free survival; and (**c**) overall survival.

When the LRFS, DFS, and OS Kaplan–Meier survival estimates were examined according to the TS_D_ groups, the following results were obtained. Five- and ten-year LRFS rates were found to be 98% and 96.6% in the TS_D_ 0–4 group, 91.6% and 87.5% in the TS_D_ 5–6 group, 94% and 94% in the TS_D_ 7–9 group, and 80.4% and 80.4% in the TS_D_ > 9 group, respectively (*p* = 0.074) (Figure 3a). Although the difference in LRFS between the groups did not reach statistical significance, it was found that survival decreased as the score increased. Five- and ten-year DFS were 96.8% and 95% in the TS_D_ 0–4 group, 88.6% and 84.7% in the TS_D_ 5–6 group, 83% and 83% in the TS_D_ 7–9 group, and 75.4% and 75.4% in the TS_D_ > 9 group, respectively (*p* = 0.064) (Figure 3b). Although the difference in LRFS between the groups did not reach statistical significance, it was found that survival decreased as the score increased. Five- and ten-year OS was 93.4% and 88.8% in the TS_D_ 0–4 group, 91.7% and 75.6% in the TS_D_ 5–6 group, 88.6% and 79% in the TS_D_ 7–9 group, and 80.2% and 63.3% in the TS_D_ > 9 group, respectively (*p* = 0.106) (Figure 3c).

**Figure 4:**
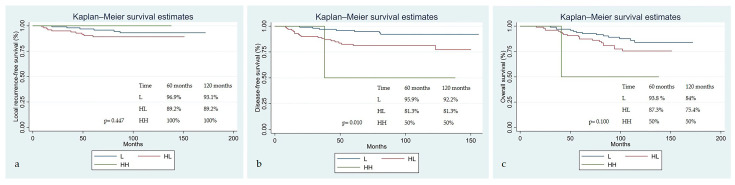
Survival according to regression pattern groups: (**a**) local recurrence-free survival; (**b**) disease-free survival; and (**c**) overall survival; L: Low–Low, HL: High–Low, HH: High–High.

When the LRFS, DFS, and OS Kaplan–Meier survival estimates were examined according to the regression pattern groups, the following results were obtained. Five- and ten-year LRFS rates were 96.1% and 93.1% in the L group and 89.2% and 89.2% in the HL group, respectively (*p* = 0.447) (Figure 4a). The 5- and 10-year disease-free survival rates were 96% and 92%, respectively, in the L group, and 81% and 81% in the HL group (*p* = 0.010). Disease-free survival was statistically significantly lower in patients with the high–low (HL) regression pattern group compared to those with the low–low (LL) group (Figure 4b). The 5- and 10-year OS rates were 93.8% and 84%, respectively, in the L group, and 87.3% and 75.4%, respectively, in the HL group (*p* = 0.100). Although the difference in OS between both TS_D_ and regression pattern groups was not statistically significant, survival decreased as the score increased (Figure 4c).

While the 60-month OS rate for the patients was 90.18%, the median Sturdza et al. 60-month estimate was 70.35%. The median Sturdza et al. survival estimate for 60 months was 70.35% (with an interquartile range of 17.1 and a range of 20.9 to 87.1). When the Sturdza et al. survival estimates were calculated for stratified FIGO stage groups, patients with stage I-II had an estimated 60 months of 77.55% (IQR = 9.5, range; 33–87.1), and those with stage III-IV had an estimated 64.8% (IQR = 15.8, range; 20.9–85.1 months). The AUC values of Sturdza et al. survival estimates (60 months: AUC = 0.456, 95% CI 0.340–0.571), TS_D_ (AUC = 0.600, 95% CI 0.493–0.706), and TS_BT_ (AUC = 0.6311, 95% CI 0.530–0.732) showed no significant difference in predicting OS (*p* = 0.159).

## 4. Discussion

Until approximately two decades ago, evidence-based planning goals for the treatment of LACC using CRT and BT were limited. Furthermore, the dose balance between EBRT and BT also varied between institutions [15,16]. Approximately twenty years ago, significant advancements were made in the design of the first prospective EMBRACE study, which was initiated in 2008. MRI was introduced for planning BT in LACC, and an adaptive target concept was defined to account for the significant regression often observed during concurrent chemotherapy with EBRT [17,18].

Chemoradiotherapy and MRI-based IGABT provide effective and consistent long-term local control in all stages of LACC, with a limited rate of severe morbidity per organ [19]. Although MRI is superior for target definition, CT-based 3D-IGABT planning is a valuable alternative option in clinical settings where more widely available methods, such as CT and ultrasound, are preferred due to logistical and financial constraints related to the limited availability of MRI [7]. Recommendations for CT-based contouring in IGABT for cervical cancer have been published by the IBS-GEC ESTRO-ABS [20].

Significant advances in radiotherapy for the definitive treatment of LACC have significantly improved locoregional control and survival. There had been no progress in the use of cisplatin for 25 years in concurrent CRT. However, new findings from studies on adjuvant chemotherapy, neoadjuvant chemotherapy, and immunotherapy have ushered in a new era of treatment [21,22,23,24].

The OUTBACK study investigated whether adjuvant chemotherapy (four cycles of paclitaxel and carboplatin) following CRT improves 5-year OS (OS). This was a multicenter, phase 3, randomized trial that included patients with FIGO 2008 stage IBI with nodal involvement or stage IB2, II, IIIB, or IVA. The use of adjuvant chemotherapy after CRT was reported not to improve OS in cervical cancer (5-year OS: 72% vs. 71%; HR, 0.9 [95% CI, 0.70–1.17; *p* = 0.81) [22]. The GCIG INTERLACE study investigated whether induction chemotherapy (carboplatin, AUC 2, and paclitaxel, 80 mg/m^2^, administered weekly for 6 weeks) before CRT improves PFS and OS. A multicenter, phase 3, randomized trial included patients with FIGO 2008 stage IB1 or IB2, IIA, IIB, IIIB, or IVA disease. Five-year OS was 80% in the induction chemotherapy group and 72% in the concurrent CRT group, *p* = 0.015; 5-year PFS: 72% and 64%, respectively, *p* = 0.013. Improved PFS and OS were reported with the use of induction chemotherapy before CRT [21]. The KEYNOTE-A18 study investigated the effect of pembrolizumab concurrently and subsequently added to CRT on PFS and OS. In a multicenter, phase 3, double-blind, randomized study, 2-year PFS was 68% in the pembrolizumab arm and 57% in the other arm, and 3-year OS was 82.6% in the pembrolizumab arm versus 74.8% (*p* = 0.004). The addition of pembrolizumab concurrently with and following CRT was reported to improve PFS and OS [24].

All these studies are discussed in terms of their different aspects. The use of older radiotherapy and BT techniques, the inclusion of patients with early-stage disease and those without para-aortic lymph nodes, the need for more extended follow-up periods, and financial toxicity are among the topics discussed. As with EBRT and BT, treatments recommended as gold standards worldwide cannot be applied to all patients. Han et al. used SEER data to evaluate 8500 women diagnosed with FIGO 2009 stage IB2-IVA cervical cancer diagnosed with EBRT between 2000 and 2020 in the United States. They reported that 64% of the 8500 women received BT in addition to EBRT, while 36% received only EBRT [25,26].

Considering all study characteristics, patient characteristics, and sources, selecting patient-specific treatments is crucial. Due to limited resources, it is necessary to choose the most suitable therapy for patients and avoid overtreatment to protect them from potential side effects [27].

Many studies have been conducted on various approaches to predict prognosis before starting treatment. The most commonly used are nomograms, which have been studied from the past to the present, utilizing clinical and radiological data, and now also including radiomic data. Studies on survival prediction using biological markers and prognosis prediction using machine learning models have been conducted [28,29,30,31,32].

In our study, we evaluated the approach of two prognosis prediction methods developed by members of a group that conducted essential studies on tumor control and side effects using concurrent CRT and MRI-guided BT in the treatment of LACC. We tested this approach in our single-center study, where we applied CT-based IGABT following concurrent CRT.

Our results showed that 60-month estimates by Sturdza et al., TS_D_ and TS_BT_, and T-score regression patterns were significantly associated with both OS and DFS outcomes. However, only TS_D_ was significantly related to local recurrence for our population. Nevertheless, 60-month OS estimates by Sturdza et al. underestimated the actual OS percentages for our population.

The nomogram by Sturdza et al. uses FIGO stage, first fraction HR-CTV volume, corpus involvement, concurrent chemotherapy, duration of treatment, age at diagnosis, and lymph node involvement parameters [12]. For our population, nearly all patients (98.04%) received concurrent chemotherapy, which significantly increased OS rates, as supported by substantial evidence from previous literature [33,34]. In addition, many of our patients were younger (84.80%), which also increases OS in our population, as well-demonstrated in previous literature [35].

These factors may partially explain why the nomogram by Sturdza et al. underestimated OS estimates for our population. However, the predictions of the nomogram by Sturdza et al. show poor performance in terms of local recurrence prediction, which may be better explained by factors outside the scope of the nomogram by Sturdza et al.

According to the analysis conducted by Schmid et al. on the EMBRACE-I study, histology, HR-CTV volume greater than 45 cm^3^, tumor necrosis, and mesorectal infiltration are additional factors explaining local failure in LACCs that received chemotherapy and MRI-guided BT [36]. According to our data, lymphadenopathy and FIGO stage are two factors significantly associated with locoregional recurrence, as determined by our survival function equality tests, which are also components of the nomogram by Sturdza et al. [12]. Additional clinical and/or MRI findings (such as uterine involvement and tumor necrosis) can improve the accuracy of the nomograms predicting local recurrence.

The T-score by Lindegaard et al. was based on MRI findings from eight anatomical regions, including the cervix, vagina, uterine corpus, parametrium (left and right), bladder, ureter, and rectum [13]. Although the scoring system solely relies on anatomical imaging findings, it was significantly associated with all oncological outcomes focused on in our study. Nevertheless, only TS_D_ was significantly related to local recurrence. In addition, T-score regression H to L was significantly associated with OS and DFS. Our results are similar to those of Lindegaard et al., who showed an association between T-score regression and oncological outcomes, including local control and OS [14]. According to our results, any model explaining oncological outcomes in LACC should include information on anatomical involvement obtained from MRI imaging.

When survival analysis was performed according to the TS_D_ and regression pattern groups, a statistically significant difference was found between the regression pattern groups for DFS. Although not reaching statistical significance between the TS_D_ groups, significantly lower scores were associated with higher LRFS, DFS, and OS. The same difference was found between the regression pattern groups for LRFS and OS.

As demonstrated by Lindegaard et al., tumor regression achieved before brachytherapy after definitive CRT in locally advanced cervical cancer is of critical importance. It not only allows for the effective application of brachytherapy but also has a significant prognostic value. Schernberg et al. studied the prognostic value of GTV reduction by measuring GTV changes between the time of diagnosis and brachytherapy in 247 patients who underwent concurrent CRT with MRI-based IGABT. Seventy-five (28%) patients had a complete response. They found a significant increase in OS, PFS, local control, and distant metastasis control in patients with a GTV reduction greater than 90% (*p* < 0.001) [37]. We use CT-based IGABT and cannot contour the GTV volume. Because the GTV is not measured in cc before and after CRT, we cannot provide a percentage reduction in tumor volume. However, when the MRIs after EBRT were re-evaluated by the radiologist for T-score determination, complete response was detected in 100 (49%) patients, and residual tumor was detected in 104 (51%) patients. Measurements of the greatest tumor diameter were obtained on the pre- and post-EBRT MRI scans, and the median primary tumor size at diagnosis was 4.4 cm (1.3–12), while the median residual tumor size after EBRT was 1 cm (0–5.3). Furthermore, when grouped according to the T-score regression pattern, 50% of our patients were in the L (Low) group, 49% in the HL (High–Low) group, and only 1% in the HH (High–High) group.

In the multicenter prospective cohort EMBRACE-I study, which investigated the effect of MRI-guided adaptive brachytherapy on local control in locally advanced cervical cancer, 5-year overall survival was reported as 74%, while in our study, 5-year overall survival was 90.8% [19]. In the EMBRACE-I study, there were 1341 patients; 1097 (81.8%) patients had squamous cell carcinoma histology, and 699 (52.1%) patients had positive lymph nodes, while in our study, 188 (92.2%) patients had squamous cell carcinoma histology, and 92 (45.1%) patients had positive lymph nodes. The EMBRACE-I study used the FIGO 2009 stage, and 693 (51.7%) patients had stage IIB, and the 5-year overall survival in this group was 78%. In our study, when the T stage was evaluated, 143 (70.1%) of the patients were stage IIB. Furthermore, a more specific group may have been selected due to the fact that 49% of patients had a complete response, the data were considered relatively low-risk, the sample size was small, and the availability of follow-up data and pre- and post-EBRT MRI images when selecting the study sample. Although these factors made our study less suitable for evaluating the impact of the T-score regression pattern, data were obtained that support the results of the study by Lindegaard et al.

Our study reflects the results of a single-center study implementing CT-based 3D-IGABT. The relatively small number of patients and the limitations inherent in a retrospective study are weaknesses. Another limitation of this study is that the patients were followed at a single institute; therefore, the findings cannot be generalized to the national and regional characteristics of patients. However, we demonstrated that TS_D_ and TS_BT_ provide essential information and may be effective in predicting survival. We believe that the TS_D_, solely obtained from pre-treatment MRI and clinical examination, can be effective in predicting prognosis and may be used in treatment selection and more widely without the data obtained during treatment (EBRT dose, BT dose, HR-CTV volume, treatment duration, etc.).

## 5. Conclusions

This study demonstrated that the nomogram by Sturdza et al. for 60-month OS predictions underestimates the survival percentages of a relatively young patient population receiving concurrent CRT. The TS_D_ obtained from the clinical examination and pre-treatment MRI scan is crucial in predicting local recurrence.

## Figures and Tables

**Table 1:** Baseline patient characteristics and treatment characteristics (*N* = 204).

Patient Characteristics		Treatment Characteristics	
Characteristics	Median (Min.–Max.) *N* (%)	Characteristics	Median (Min.–Max.) *N* (%)
Age (years)	52 (29–89)	EBRT technique 3D-CRT IMRT/VMAT	112 (54.9%) 92 (45.1%)
KPS score 100 90 80 70	100 (70–100) 134 (65.7%) 59 (28.8%) 10 (4.9%) 1 (0.5%)	Total EBRT doses (Gy)	50 (45–64.4)
		Total EBRT fractions	25 (23–30)
Pre-treatment hemoglobin (g/dL)	11.85 (7.2–15.2)	Brachytherapy dose per fraction (Gy)	6.35 (5–8.5)
Menopause status Postmenopause Premenopause Perimenopause	117 (57.4%) 76 (37.3%) 11 (5.4%)	Brachytherapy fraction number	4 (2–6)
Pathology Squamous cell carcinoma Adenocarcinoma Adenosquamous carcinoma Serous papillary carcinoma Mucinous papillary carcinoma	188 (92.2%) 12 (5.9%) 2 (1%) 1 (0.5%) 1 (0.5%)	HR-CTV volume cc	30.8 (4.33–111.78)
		HR-CTV volume group ≤30 cc >30 cc	98 (48.04%) 106 (51.96%)
Involved lymph node Yes No	92 (45.1%) 112 (54.9%)	HR-CTV D90 EQD2 (Gy)	81.65 (70.1–97.2)
Involved lymph node region Pelvic region Pelvic + para-aortic region Para-aortic region No	79 (38.7%) 11 (5.4%) 2 (1%) 112 (54.9%)	Total treatment time group ≤80 days >80 days	105 (51.47%) 99 (48.53%)
FIGO 2018 Staging IB2 IB3 IIA2 IIB IIIA IIIB IIIC1 IIIC2 IVA	6 (2.9%) 4 (2%) 2 (1%) 84 (41.2%) 3 (1.5%) 11 (5.4%) 76 (37.3%) 13 (6.4%) 5 (2.5%)	Concurrent chemotherapy drugs Cisplatin Carboplatin Low dose paclitaxel–carboplatin No	198 (97.1%) 1 (0.5%) 2 (1%) 3 (1.5%)
		Concurrent chemotherapy cycles	5 (0–7)
FIGO 2018 Staging Group Stage I-II Stage III-IV	96 (47.1%) 108 (52.9%)	60-month estimates by Sturdza et al.	70.35 (20.9–87.1)
FIGO 2018 T Staging IB2 IB3 IIA1 IIA2 IIB IIIA IIIB IVA	11 (5.4%) 8 (3.9%) 4 (2%) 2 (2%) 143 (70.1%) 4 (2%) 27 (13.2%) 5 (2.5%)	TS_D_	5.5 (1–16)
		TS_BT_	1 (0–6)
FIGO 2018 T Staging Group Stage I-II Stage III-IV	168 (82.4%) 36 (17.6%)	Regression pattern L HL HH	102 (50%) 100 (49.02%) 2 (0.98%)

KPS: Karnofsky performance scale; FIGO: The International Federation of Gynecology and Obstetrics; EBRT: external beam radiation therapy; 3D-CRT: three-dimensional conformal radiation therapy; IMRT: intensity-modulated radiation therapy; VMAT: volumetric modulated arc therapy; Gy: gray; HR-CTV: high-risk clinical target volume; HR-CTV D90: minimum dose delivered to 90 of the high-risk clinical target volume; EQD2: equivalent dose in 2 Gy fractions; TS_D_: pre-treatment T-score at diagnosis; TS_BT_: pre-treatment T-score at brachytherapy; L: Low–Low; HL: High–Low; HH: High–High.

**Table 2:** Survival function equality test for oncological outcomes.

Variable	Local Recurrence-Free Survival *p*-Value	Disease-Free Survival *p*-Value	Overall Survival *p*-Value
Age ≥ 65 years	0.341	0.591	0.367
Pathology type	0.604	0.247	0.209
Regression pattern	0.448	0.010	0.101
Pelvic and/or para-aortic lymphadenopathy	0.031	0.988	0.665
FIGO 2018 stage group	0.024	0.965	0.755
FIGO 2018 T stage group	0.623	0.528	0.046
Pelvic radiotherapy technique	0.377	0.080	0.281
HR-CTV volume ≥ 30 cc	0.561	0.474	0.347
Treatment duration (>80 days)	0.478	0.435	0.879

**Table 3:** Univariate Cox regression analysis.

	Local Recurrence-Free Survival	Disease-Free Survival	Overall Survival
Variable	HR (95% CI), *p*-Value	HR (95% CI), *p*-Value	HR (95% CI), *p*-Value
Age	0.977 (0.933–1.022), 0.312	1.001 (0.968–1.034), 0.970	1.018 (0.988–1.048), 0.241
KPS score (0–100)	1.026 (0.937–1.123), 0.584	0.995 (0.935–1.058), 0.862	0.992 (0.938–1.049), 0.771
Hemoglobin level (g/dL)	1.015 (0.730–1.412), 0.930	1.006 (0.786–1.288), 0.962	0.962 (0.764–1.211), 0.741
Menopause status (compared to peri)			
Post	0.959 (0.123–7.499), 0.969	0.924 (0.215–3.976), 0.915	2.909 (0.394–21.498), 0.295
Pre	0.702 (0.082–6.014), 0.747	0.421 (0.085–2.090), 0.290	1.420 (0.177–11.380), 0.741
Pathology (compared to SCC)	1.477 (0.336–6.497), 0.606	1.855 (0.641–5.370), 0.255	1.820 (0.704–4.703), 0.217
Presence of lymphadenopathy	3.633 (1.035–12.748) 0.044	1.006 (0.471–2.149), 0.988	0.860 (0.434–1.703), 0.665
FIGO 2018 T stage group (compared to I-II)	0.691 (0.157–3.043), 0.625	1.338 (0.540–3.317), 0.529	2.081 (0.995–4.354), 0.052
FIGO 2018 group (compared to I-II)	3.413 (1.101–10.583), 0.033	0.983 (0.462–2.092), 0.965	0.899 (0.459–1.761), 0.756
3D-CRT (compared to IMRT/VMAT)	1.606 (0.556–4.634), 0.381	2.126 (0.896–5.043), 0.087	1.500 (0.713–3.153), 0.285
HR-CTV volume	1.022 (0.995–1.050), 0.114	1.017 (0.995–1.039), 0.140	1.010 (0.986–1.034), 0.423
HR-CTV of EQD2 D90 (Gy)	1.030 (0.936–1.133), 0.543	1.000 (0.928–1.077), 0.998	1.002 (0.938–1.071), 0.947
Treatment duration (in months)	1.007 (0.982–1.033), 0.577	1.006 (0.986–1.026), 0.585	1.007 (0.990–1.025), 0.438
60-month estimates by Sturdza et al.	0.993 (0.959–1.027), 0.670	0.973 (0.950–0.997), 0.028	0.977 (0.957–0.997), 0.025
TS_D_	1.184 (1.002–1.400), 0.047	1.193 (1.052–1.354), 0.006	1.126 (1.001–1.265), 0.048
TS_BT_	0.997 (0.670–1.484), 0.988	1.297 (1.037–1.622), 0.023	1.339 (1.096–1.637), 0.004
Regression group (compared to L)			
HL	1.850 (0.672–5.091), 0.234	3.090 (1.298–7.356), 0.011	1.795 (0.892–3.612), 0.101
HH	N/A; N/A	8.072 (0.990–65.784), 0.051	5.004 (0.653–38.315), 0.121

HR: hazard ratio; CI: confidence interval; KPS: Karnofsky performance scale; SCC: squamous cell carcinoma; 3D-CRT: three-dimensional conformal radiotherapy; IMRT: intensity-modulated radiotherapy; HR-CTV: high-risk clinical target volume; EBRT: external beam radiotherapy; HR-CTV D90: 90% of the high-risk clinical target volume; EQD2: equivalent dose in 2 Gy fractions; Gy: gray; TS_D_: pre-treatment T-score at diagnosis; TS_BT_: pre-treatment T-score at brachytherapy; HL: High–Low; HH: High–High.

**Table 4:** Multivariate Cox regression models for local recurrence-free survival.

Variable	Stratification	HR (95% CI)	*p*-Value
TS_D_	Continuous	1.203 (1.021–1.417)	0.027
Lymphadenopathy	Yes vs. no	3.968 (1.128–13.957)	0.032

HR: hazard ratio; CI: confidence interval; TS_D_: pre-treatment T-score at diagnosis.

## Data Availability

The raw data supporting the conclusions of this article will be made available by the authors on request.

## Abbreviations

The following abbreviations are used in this manuscript:

3D-CRT	Three-dimensional conformal radiotherapy
BT	Brachytherapy
CRT	Chemoradiotherapy
CT	Computed tomography
DFS	Disease-free survival
EBRT	External beam radiotherapy
EQD2	Equivalent dose of 2Gy
FIGO	International Federation of Gynecology and Obstetrics
Gy	Gray
HL	High–Low
HR	Hazard ratio
HR-CTV D90	90% of the high risk-clinical target volume
IGABT	Image-guided adaptive brachytherapy
IMRT	Intensity-Modulated Radiation Therapy
IQR	Interquartile range
KPS	Karnofsky performance scale
LL	Low–Low
LRFS	Local recurrence-free survival
MRI	Magnetic resonance imaging
OS	Overall survival
SCC	Squamous cell carcinoma
TSBT	Pre-treatment T-score at brachytherapy
T-score	Tumor score
TSD	Pre-treatment T-score at diagnosis
